# The role of guideline organizations in nationwide guideline implementation: a qualitative study

**DOI:** 10.1186/s12961-024-01253-0

**Published:** 2024-12-23

**Authors:** Andrea C. Thoonsen, Hanneke Merten, Toby T. Broeders, Anika Gans, Ilse van Beusekom, Diana M. J. Delnoij, Martine C. de Bruijne

**Affiliations:** 1 Amsterdam UMC, Department of Public and Occupational Health, Amsterdam Public Health Research Institute, Vrije Universiteit Amsterdam, Van Der Boechorststraat 7, NL-1081 BT Amsterdam, The Netherlands; 2 Zorginstituut Nederland, Department of Care, Willem Dudokhof 1, NL-1112 ZA Diemen, The Netherlands; 3 Erasmus School of Health Policy & Management, Department of Health Care Governance, Erasmus Universiteit Rotterdam, Burgemeester Oudlaan 50, Rotterdam, The Netherlands

**Keywords:** Clinical practice guidelines, Guideline adherence, Implementation science, Guideline organizations, Quality improvement

## Abstract

**Background:**

Research indicates suboptimal uptake and impact of clinical practice guidelines in Dutch healthcare. Dutch guideline organizations, i.e. guideline developers, governmental agencies, health insurers and other national organizations, develop, authorize and/or support the use of guidelines in Dutch clinical practice. These organizations influence the end users’ awareness, accessibility, understanding, acceptability and applicability of guidelines and, therefore, play a crucial role in guideline implementation. This study explores how they plan, execute, monitor and evaluate guideline dissemination and implementation.

**Methods:**

Utilizing a qualitative design, we conducted semi-structured interviews with 35 participants from 24 guideline organizations. We conducted framework analysis, using theories on guideline implementation planning, the ‘taxonomy of strategies for achieving guideline implementation and compliance’ and the principles of logic models to analyse the data.

**Results:**

Most guideline organizations made limited use of implementation planning approaches that are known to enhance guideline uptake and impact. These approaches include pre-identifying implementation barriers, engaging stakeholders and applying implementation theories, models and frameworks to select and tailor implementation strategies. Instead, they primarily relied on a standard set of predominantly dissemination and occasional implementation strategies known to be practical in terms of ease, cost and time. Commonly used implementation strategies included distributing, advertising and presenting guideline materials, along with providing additional implementation supporting materials. Regarding monitoring and evaluation methods, few organizations assessed the process, outcome or impact of guideline implementation. Those that did primarily relied on clinical peer review and benchmark information for their assessments.

**Conclusions:**

While Dutch guideline organizations recognized and endorsed the importance of implementation, this did not consistently translate into tailored implementation actions. Most guideline organizations did not have an integrated, structural and well-thought-out plan for implementation. The lack of regular, structured monitoring and evaluation raised uncertainties about the effectiveness of implementation in supporting end users and improving patient outcomes. Suggested follow-up research and practice enhancements could strengthen central-level implementation efforts, fostering more effective local implementation and, ultimately, improving health outcomes.

**Supplementary Information:**

The online version contains supplementary material available at 10.1186/s12961-024-01253-0.

## Background

Clinical practice guidelines are vital tools for optimizing patient care [1]. They provide recommendations based on a systematic review of the available scientific evidence and an evaluation of the benefits and harms of alternative care options [1]. These guidelines help healthcare professionals and patients in making informed medical decisions by promoting interventions of proven benefit (appropriate care) and discouraging ineffective or potentially harmful practices (low-value care) [2, 3]. Guidelines have shown the potential to support appropriate care delivery and reduce treatment inequality [3, 4]. They serve as valuable tools in managing healthcare challenges such as rising expenditures, population ageing, constrained resource availability and its negative environmental impact [3–5].

In the Netherlands, guidelines have formed the backbone of quality policies concerning prevention, diagnosis, treatment and healthcare organization for years [6]. Since the start of the development of Dutch guidelines in 1982, they offer substantive guidance not only for delivering patient care but also for education, clinical peer review, local protocols and quality systems, initiatives and registrations [6, 7]. Guidelines are also increasingly used by governmental agencies to enhance cost-effectiveness, regulate the quality of care and establish financing agreements [6].

The value of guidelines depends not only by the quality and accuracy of their recommendations but also by their successful implementation in clinical practice [8, 9]. Dutch law expects healthcare facilities and professionals to act in accordance with established guidelines, though justifiable deviations are possible [10]. Hence, the primary responsibility for implementing these guidelines and reshaping clinical practice and organizational structures rests with them.

As of 2024, approximately 2200 guidelines were available for medical specialists, 350 for nurses, 140 for general practitioners and 50 for mental health professionals [11–14]. These numbers continue to increase, while research indicates that their implementation in local policy and practice is unpredictable, slow, complex and suboptimal [15–20]. Consequently, the quality of patient care may stay behind, and valuable healthcare resources may be wasted. It is important to recognize that just publishing guidelines and expecting healthcare professionals and facilities to implement them does not guarantee uptake or improvements in practice [21].

Beyond healthcare professionals and facilities, which are the primary guideline end users, other important players fulfil a vital role in guideline implementation: guideline organizations. This broadly conceived term refers to a diverse group of centrally operating national organizations that, as part of their responsibilities, are tasked with developing guidelines, authorizing them and/or supporting their use in Dutch clinical practice. It includes scientific/professional organizations, patient organizations, governmental agencies, health insurers and other national (umbrella) organizations. Examples of Dutch guideline organizations are the Dutch College of General Practitioners (NHG) and the National Health Care Institute (ZIN). These organizations can influence the end users’ awareness, accessibility, understanding, acceptability and applicability of guidelines [22–24]. They can stimulate implementation by end users in local clinical practice through using tailored dissemination and implementation strategies, addressing implementation barriers and optimizing facilitators [24–26].

The optimal approach for guideline organizations to accelerate the implementation process has received limited attention in research [22, 27, 28]. The envisioned roles and responsibilities of guideline organizations in implementation are briefly outlined in the Dutch ‘guideline for guidelines’ – the AQUA guideline [29]. This document provides recommendations for the development, revision and implementation of Dutch guidelines. It emphasizes raising guideline awareness and addressing national barriers where possible and suggests several implementation planning, execution, monitoring and evaluation approaches [29].

It is unclear how guideline organizations actually approach their implementation role in practice. Examining their approaches could help identify points of improvement. This could facilitate the development of enhanced guideline implementation policies and practical guidance, strengthening implementation efforts at the national level. This can serve as a foundation for more effective implementation at the local, healthcare professional and facility level, ultimately improving health outcomes. Thus, the aim of this study is to explore how Dutch guideline organizations plan and execute dissemination and implementation strategies and how they monitor and evaluate the implementation process, outcomes and impact of their guidelines.

### Methods

The consolidated criteria for reporting qualitative research (COREQ) were used for reporting the methods and findings (Additional file 1) [30].

### Study design

We used a qualitative design with semi-structured interviews to gain in-depth insight into the implementation approaches of guideline organizations.

The current study is part of a larger qualitative interview project, in which we also investigated implementation barriers and facilitators encountered by Dutch guideline organizations. The results pertaining these aspects are addressed elsewhere.

### Participants

Guideline organizations were eligible if they were scientific/professional organizations, knowledge institutes, governmental agencies, health insurers, patient organizations; or other national (umbrella) organizations that developed guidelines, published them and/or actively supported their use in Dutch clinical practice. We recruited representatives from guideline organizations based on their insights into their organization’s role or direct involvement in implementing guidelines. We used purposive sampling methods to recruit a broad sample of these representatives. We recruited potential participants through contact information from guideline organization websites and through contacts of the research team. Furthermore, representatives who were interviewed were asked if they knew additional representatives who could participate (snowball sampling). Participants were contacted via email or telephone. Before agreeing to participate, participants received information letters containing study background, privacy and informed consent information.

### Data collection

Interviews, conducted between March and October 2023, were held via videoconference or in-person based on participant preference. Interviews were held in Dutch by one or two researchers (T.B., A.G. and A.T.), who had a background in health policy studies, were trained in interviewing techniques and had interview experience. We used a semi-structured approach with an interview guide (Additional file 2) with primarily open-ended questions, while at the same time providing the possibility for a more in-depth exploration of certain topics. The interview guide was structured based on the aforementioned theoretical constructs and covered various aspects, including (1) implementation planning approaches, (2) dissemination and implementation strategies, (3) methods for implementation process, outcomes and impact monitoring and evaluation and (4) more general inquiries, such as implementation barriers and facilitators and opportunities for improvement.

Each interview started with asking participants for permission to record for transcription. Written and verbal informed consent were obtained. Interviews ranged from 30–100 min, and participants received an interview summary for member checking afterwards. Data collection continued until no new themes emerged, signifying data saturation.

### Theories, taxonomies, models and frameworks

We used the AQUA guideline as a frame of reference to assess how actual implementation approaches aligned with the envisioned roles and activities. We also compared the implementation practices with established scientific theories and taxonomies on guideline implementation, using various theoretical constructs to guide the interviews and data analysis.

### AQUA guideline – Dutch ‘guideline for guidelines’

In the AQUA guideline [29] Dutch guideline experts have outlined how high-quality guidelines should be developed. The process follows three phases: preparation, development and finalization.

In the preparation phase, the guideline topic, purpose, and target audience of the guideline are established. It involves forming a guideline committee, which includes relevant scientific and professional organizations, patient groups and methodological experts. The involved methodological experts should also have a basic understanding of implementation. Additional stakeholders, such as governmental agencies and health insurers, may also be invited to contribute.

The development phase begins with a problem analysis, in which the experienced challenges in healthcare (in terms of practice and organization of care) are identified. Based on the resulting key questions, the existing knowledge is systematically reviewed and presented in a transparent manner, preferably following the GRADE methodology [31, 32]. When developing the guideline recommendations, factors such as health benefits, side effects, safety, cost-effectiveness/budget impact and the preferences of patients and professionals should be considered, along with implementation barriers and facilitators. The acceptability and feasibility of the recommendations should also be taken into account. Additionally, guidelines should propose methods for monitoring and evaluating care, such as quality indicators. Implementation supporting materials, such as protocols, decision aids and educational materials, should also be made available to facilitate self-management and shared decision-making.

According to the AQUA guideline, the finalization of the guideline starts with a pre-publication commentary phase, in which end users and guideline organizations are consulted through written commentary rounds, surveys or pilot implementations to evaluate the content and applicability of the guideline. In the authorization phase, the guideline must be approved by all primary involved professional groups and patient organizations, before it officially becomes part of the professional standard. The guideline includes a maintenance procedure detailing responsibilities and timelines for updates. After publication, the guideline organizations involved should be committed to actively promoting the guideline’s practical application. They should raise awareness of the guideline and, to their extent possible, address any national barriers to its implementation. If certain implementation preconditions need to be met first, a timeline and allocation of responsibilities should be specified, so that it is clear when implementation of the guideline can be expected. The AQUA guideline states that implementation should be carried out by healthcare facilities, professionals and other local stakeholders. The guideline should facilitate implementation by listing barriers and facilitators, as well as necessary preconditions and recommendations for implementation. The AQUA guideline links to an implementation checklist [33] that supports the identification of potential barriers to effective implementation. Lastly, it recommends to document implementation strategies in an implementation plan.

### Theories on implementation planning

In terms of implementation planning, previous research suggests that selecting and tailoring implementation strategies using three specific approaches can enhance the uptake and implementation of guidelines [24–26, 34, 35]. Dissemination and implementation strategies that are (1) aligned with stakeholder preferences and (2) designed to address existing barriers are more likely to improve professional practice [25, 26, 34]. Third, applying principles from implementation theories, models and frameworks (TMFs) to guide and shape these strategies can further enhance implementation outcomes [24]. In our investigation of implementation planning approaches, we, therefore, conducted an in-depth exploration of whether guideline organizations used pre-identified barriers, stakeholder engagement and implementation TMFs.

### Classifying dissemination and implementation strategies

In our exploration of executed dissemination and implementation strategies, we applied the ‘taxonomy of strategies for achieving guideline implementation and compliance’ of Mazza et al. [36], later expanded by Gagliardi and Alhabib [37]. This taxonomy allowed us to examine strategies and classify them into five domains: professional, patient/consumer, financial, organizational and regulatory.

### Classifying monitoring and evaluation methods

To systematically analyse the implementation monitoring and evaluation methods used by guideline organizations, we categorized these methods into three domains – process, outcomes and impact – based on the principles of logic models [38]. In this study, process monitoring and evaluation refers to methods used to assess implementation activities (outputs) and the actual use of guidelines. Outcome monitoring and evaluation involves methods for evaluating short- and medium-term outcomes, such as changes in end-user practice (behaviour, knowledge and skills). Impact monitoring and evaluation refers to methods that assess long-term changes related to achieving desired goals, including improvements in health outcomes, system performance and societal benefits.

### Data analysis and synthesis

All interviews were audio recorded and transcribed verbatim. Interview transcripts were analysed using the principles of framework analysis [39]. Both deductive and inductive coding were used. For deductive coding, the researchers (A.T., T.B. and A.G.) developed an initial codebook, based on the aforementioned theoretical constructs. In addition, open coding was used to include interesting themes that emerged from the data. Throughout the coding process, the codebook was updated iteratively. Initially, two researchers (A.T. and either T.B. or A.G.) independently coded the first eight interviews to align coding. Subsequently, one researcher (T.B., A.G. or A.T.) coded the rest, cross-checked by a second researcher (A.T. or H.M.). The final coding tree is provided in Addtional file 3. Coding was conducted using MAXQDA (version 2022).

We used in-text quotes (translated from Dutch) to highlight key themes or perspectives that were particularly representative, significant or illustrative or where interviewee voices added clarity or emphasis. In the results tables, we systematically provided quotes for all identified implementation planning, execution, monitoring and evaluation activities.

### Ethical considerations

The Medical Ethics Committee of Amsterdam UMC reviewed the study protocol and decided that the study did not fall under the scope of the Dutch Medical Research Involving Human Subjects Act, since the participants were not subject to medical procedures nor required to follow rules of behaviour [40]. Therefore, no further formal ethical approval was required (statement ID 2021–0417). As previously stated, all participants signed an informed consent form before the start of the interview and received an interview summary for member checking afterwards. The transcripts and audio recordings were stored and analysed in accordance with the General Data Protection Regulation.

## Results

### Participant characteristics

A total of 35 participants from 24 different guideline organizations were interviewed. An overview of the participants and their respected guideline organizations can be found in Table 1. To ensure participant confidentiality, they were categorized into four distinct groups: guideline developers (scientific/professional organizations and knowledge institutes), governmental agencies, health insurers and other national organizations. Findings are presented using these group terms, but when results apply to multiple groups, the broader term ‘guideline organizations’ is used. Eight participants held dual roles, such as simultaneously working at a scientific organization and a knowledge institute or serving as medical specialists while also being employed at a scientific organization.

**Table 1:** Interview participant characteristics

Categories of guideline organizations	Guideline organizations	Number of participants
Guideline developers (scientific/professional organizations and knowledge institutes)	Dutch Quality Alliance in Mental Health Care (Akwa GGZ)	1
	Netherlands Comprehensive Cancer Organisation (IKNL)	1
	Knowledge Institute of the Dutch Association of Medical Specialists (KIMS)	2
	Dutch College of General Practitioners (NHG)	1
	Dutch Society for Internal Medicine (NIV)	2
	Dutch Society of Physicians for Respiratory Diseases and Tuberculosis (NVALT)	1
	Dutch Geriatrics Society (NVKG)	1
	Dutch Society of Obstetrics and Gynaecology (NVOG)	2
	Netherlands Society of Cardiology (NVVC)	1
	Dutch Society for Surgery (NVvH)	1
	Netherlands Association of Nurses and Care Workers (V&VN)	2
	Netherlands Association of Sports Medicine (VSG)	2
Governmental agencies	Health and Youth Care Inspectorate (IGJ)	1
	Dutch Healthcare Authority (NZa)	1
	Ministries of Public Health, Welfare and Sport (VWS)	2
	National Health Care Institute (ZIN)	4
	Netherlands Organisation for Health Research and Development (ZonMw)	1
Health insurers	Health insurance company	1
	Health Insurers Netherlands (ZN)	1
Other national organizations	Netherlands Federation of University Medical Centres (NFU)	1
	Dutch Hospital Association (NVZ)	1
	Netherlands Patients Federation (PFN)	1
	Health Care Evaluation and Appropriate Use (ZE&GG)	2
	Independent Clinics Netherlands (ZKN)	2

### Implementation planning approaches

Guideline organizations affirmed that the primary responsibility for implementing guidelines rests with healthcare professionals and facilities, who, in consultation with patients, determine clinical practices. Yet, they recognized that these guideline end users could not manage implementation alone and required support from guideline organizations. Consequently, they facilitated implementation through various approaches and to varying extents.

Guideline developers were the most actively involved in implementation as they had ownership over the guidelines and contributed the majority of members to guideline committees. These committees, regularly assisted by an implementation advisor, also often handled the planning and development of dissemination and implementation strategies. Only a few guideline organizations had an additional, dedicated implementation team.

Guideline organizations employed diverse implementation planning approaches, selecting their dissemination and implementation strategies through various methods and tailoring them to varying degrees. Table 2 provides an overview of the different implementation planning approaches along with exemplary quotes. Guideline organizations prioritized implementation strategies for urgent guideline topics, such as prevalent health issues, over comparatively ‘smaller’ guideline topics.

**Table 2:** Implementation planning approaches used by the guideline organizations

Implementation planning approaches	Exemplary quotes
Pre-identification of barriers (and facilitators)	“… *And then you can also pre-identify facilitating and hindering factors. As a [guideline organization], we strive to address the hindering factors at a more macro level.*” – Interviewee 1, other national organization
Development of implementation plans	“*Then we have an implementation table that needs to be filled out. In it, you specify, for example, the timeline for implementation, the expected cost effects, implementation prerequisites, potential barriers, any actions that need to be taken for implementation, the assignment of responsibilities, and the ability to include various comments.*” – Interviewee 10, guideline developer
Engagement of stakeholders (e.g. through commentary rounds, focus groups, surveys, interviews)	“*Then we engage in discussions with potential users, asking them questions such as: what do you need? What works, and what doesn’t? We did that through interviews.*” – Interviewee 1, other national organization
Use of implementation TMFs (e.g. Grol and Wensing Implementation of Change model [41], MIDI model[42])	“*We did use an implementation model by Grol and Wensing, to have some kind of evidence to support our approach. It is a framework that is commonly used.*” – Interviewee 12, other national organization
Stakeholder analysis (e.g. analysis of knowledge, attitude, behaviour or power/interest matrix)	“*So, based on all that information, you create a matrix with all the interests of all parties, and you can take them into account during those meetings.*” – Interviewee 6, governmental agency
Guideline committee’s motivations and preferences to conduct certain implementation strategies determine the final selection of strategies	“*Well, one thing is whether a guideline committee member wants to do it. I think that’s actually quite important. For example, if I look at a case involving educational materials, if no one feels inclined to create or write it, then it won’t be made, because I’m not going to do it. I don’t have the profound expertise.*” – Interviewee 15, guideline developer
The practicality of specific implementation strategies (e.g. ease of execution) and the availability of implementation resources (e.g. funding, time) determine the selection of specific implementation strategies	“*We’ve been very practically addressing [implementation]. And for now, this seemed like the most low-hanging fruit, the easiest, and the cheapest to do. But we didn’t look further than that, no.*” – Interviewee 34, guideline developer
The selection of specific implementation strategies is made in collaboration with a marketing agency, implementation consultancy, communication advisor or educational specialist	“*So, these are often, well, three to five [healthcare professionals] who, together with a marketing agency we collaborate with, sit down and essentially discuss what kind of implementation product it will be.*” – Interviewee 3, guideline developer
Replication/adaptation of (successful) implementation strategies of other guideline organizations/similar projects	“*Well, what we actually did is we asked other guideline organizations for examples. And some really set up entire podcasts and others create some kind of poster and some send entire quizzes via email.*” – Interviewee 34, guideline developer
Guideline implementation pilot testing	“*During the development phase or when your concept is ready, we aim to carry out a pilot implementation, during which we assess the feasibility and practicability of adhering to the recommendations.*” – Interviewee 3, guideline developer
No structured implementation planning approach (e.g. focus on doing instead of planning/selecting/tailoring)	“*But at the end of the day you have to get started. … We learn the most by just starting.*” – Interviewee 6, governmental agency

Most guideline organizations barely tailored their implementation strategies through pre-identifying barriers, engaging stakeholders or utilizing implementation TMFs. They often employed a standard set of implementation strategies that they had used before and that had proven to be practical in terms of ease, cost and time. Limited implementation funds, time constraints, and insufficient implementation knowledge and motivation within guideline committees hindered strategy selection and tailoring:

“*There is substantial brainstorming about creating podcasts, webinars and micro-learning quizzes, all these methods to get guidelines to the user. … However, this often depends heavily on the commitment of guideline committee members. Whether such ideas actually materialize depends on factors like budget and time availability. But very often, it seems like voluntary work from the committee members to engage in these extra activities.*” – Interviewee 18, guideline developer.

Several guideline organizations pre-identified guideline-specific implementation barriers (and facilitators). However, they did not consider these insights in the next crucial step: selecting and tailoring their implementation strategies to effectively address these barriers. They primarily used two methods for pre-identifying barriers: implementation determinant analysis or integrating barrier pre-identification into the development of an implementation plan. Guideline committees commonly created these plans after formulating the guideline recommendations, outlining implementation timeline, expected cost effects, prerequisites, potential barriers, actions required for implementation and organizations responsible for those actions. However, guideline organizations stated that the quality of these implementation plans frequently fell short. Plans were hastily developed at the end of the guideline development process, with insufficient time allocated. Furthermore, committee members frequently struggled with completing these plans, as they involved aspects of healthcare organization beyond their expertise. Occasionally, implementation plans were copied from previous guidelines. These issues resulted in very concise plans lacking specificity. Moreover, guideline organizations acknowledged a lack of adequate attention and action to the plans during the follow-up phase, encompassing the practical elaboration and execution of dissemination and implementation strategies:

“*… You’re hitting the nail on the head because it’s exactly what we’re bothered with. What implementation plan did we write in the previous guideline? Oh, let’s adjust the titles a bit. We rearrange a few words, and voila, we’ve delivered the implementation plan. But the follow-up? None. And that’s just a shame, really.*” – Interviewee 21, guideline developer.

Regarding stakeholder engagement, numerous stakeholders played various roles in the implementation planning. As previously mentioned, the guideline committee, mainly comprising of relevant healthcare professionals and patients, actively engaged in developing the guideline and its implementation plan. Depending on the guideline’s topic, other guideline organizations (e.g. governmental agencies or health insurers) were invited to contribute, either through mandated representation in the guideline committee or commenting on the content and applicability (e.g. organizational, financial or legal) of the guideline and its implementation plan in the pre-publication commentary phase. However, other guideline organizations did not consistently comment on the guidelines, for example, due to limitations in their capacity to review the large number of guidelines.

“*… this task is so extensive that it’s not feasible to respond to all guidelines as they are often thick stacks of paper. We try to prioritize the main issues.*” – Interviewee 25, health insurer.

A small number of guideline organizations engaged stakeholders post commentary and publication. Some guideline developers sporadically evaluated needs or conducted pilot testing with healthcare professionals, patients and their relatives to identify implementation barriers or develop implementation strategies (e.g. implementation tools). Methods of engagement involved for example focus groups, surveys or interviews.

When asked about using implementation TMFs in the implementation planning phase, nearly all guideline organizations stated they did not explicitly use them. However, these theoretical constructs could play an indirect role, as they were occasionally integrated into implementation guides and operational action plans that a few guideline organizations used. Within these instances, participants often cited the influence of the Grol and Wensing Implementation of Change model [41].

### Dissemination and implementation strategies

Guideline organizations used a large variety of different dissemination and implementation strategies as outlined in Table 3. Not all strategies mentioned by the interviewees fit the predefined strategies in the modified taxonomy of Mazza et al. [36]. These strategies were classified and added. Furthermore, we were unable to classify all implementation strategies within the existing five domains of the taxonomy. Several strategies were developed and applied by guideline organizations at the national level, aiming to change the practice of other guideline organizations (macro level) and/or healthcare facilities (meso level), rather than targeting groups or individual healthcare professionals. To account for these broader, system-level strategies, we introduced a new domain, ‘central’, in addition to the existing domains. While many strategies concentrated on the professional or central domain, fewer were identified in the patient, regulatory or financial domain. Guideline organizations did not address strategies within the organizational domain.

**Table 3:** Dissemination and implementation strategies employed by the guideline organizations

Categories of implementation strategies (classified by Mazza et al. [36], expanded by Gagliardi and Alhabib [37])^a^	Implementation strategy (details)	Exemplary quotes
**1. Professional **		
Distribute guideline materials	Distribute draft guideline to other guideline organizations and healthcare professionals for feedback on the content and applicability (e.g. organizational, financial or legal) of the guideline and its implementation plan (pre-publication commentary phase)	“*When the guideline is nearly finished, you enter the commentary phase. [The guideline] is then communicated to the members via email and the website.*” – Interviewee 32, guideline developer
	Mass emailing final guideline	“*As a standard practice when something new comes up, we include it in the newsletter and mass email.*” – Interviewee 30, guideline developer
Provide feedback guideline compliance data and information	Audit and feedback benchmark information for healthcare professionals and facilities (e.g. Transparency Calendar [43], Implementation Monitor [44] or health insurer claims data)	“*We’ve noticed that you can tempt hospitals the most by benchmark information, with which you demonstrate: here is where your hospital stands compared to other hospitals in the Netherlands.*” – Interviewee 28, health insurer
Enable self-audit	Provide indicators to healthcare facilities for self-audit and feedback	“*We also create indicators, primarily meant for self-assessment purposes only. It’s about preventing them from being included solely in outcome-based financing.*” – Interviewee 2, guideline developer
Present guideline materials at meetings	Present guideline materials at meetings (e.g. conferences or annual meetings)	“*That also happens, you know, that the outcomes of such a study and the guideline are discussed at a congress*” – Interviewee 30, guideline developer
Educate groups about guideline intent/benefits	Education through workshops, webinars, e-learnings, micro learning quizzes, instruction videos	“*… Participants receive an email with questions focused on the core recommendations from the guidelines. That they work just a few minutes on enhancing their knowledge about the guideline.*” – Interviewee 3, guideline developer
Advertise guideline materials	Book publications, journal publications, newsletters, website publications, media releases/campaigns, social media releases, podcasts	“*… We also have a newsletter, a magazine where occasional updates about new guidelines are included, and a LinkedIn page.*” – Interviewee 22, guideline developer
	Submit guideline to database (e.g. Guideline Database [45], Register [46])	“*And that it’s published on the guideline database. It’s actually a kind of implementation tool, as specialists can look up what the recommendation is.*” – Interviewee 10, guideline developer
	Provide app with guideline content	“*We have an app called [app name], and in that app, there’s also, for example, a summary and summary cards, and it also includes a formulary.*” – Interviewee 15, guideline developer
	Publish drug formulary	“*We also develop a formulary.*” – Interviewee 2, guideline developer
*Provide additional implementation supporting materials*^b^	Implementation toolkit, patient pathways, visuals, conversation guides, sample presentations, clinical decision support tools, guideline summary/factsheet, pocket cards, triage guide, infographics, practice manuals	“*So, we create summary cards for all guidelines, featuring core recommendations within a two-page summary.*” – Interviewee 29, guideline developer
*Clinical peer review*	Guideline developers conduct periodic clinical peer reviews of care departments (required for maintaining registration for certain healthcare professionals)	“*… I know that within clinical peer review, questions are asked about whether and how guidelines have been implemented in daily practice.*" – Interviewee 21, guideline developer
*Recruit champions*	Recruit champions who recommend, support and stimulate implementation	“*… That is often an expert in a specific field – these could be physicians, but also specialists or nurses – who possess substantial knowledge about a topic and are willing to take a stand to ensure that the field moves toward the recommended course of action.*” – Interviewee 1, national organization
**2. Patient/consumer**		
*Advertise guideline*	Patient organization website publication	“*The patient organizations also publish, they also mention: a guideline has been updated. They explain what’s new in a guideline.*” – Interviewee 27, national organization
	Patient website publication (e.g. www.thuisarts.nl)	“*We’ve set up governance agreements. … That since 2016, for secondary care guidelines, patient information is always created and made available on Thuisarts.*” – Interviewee 27, national organization
	Patient version of guideline/patient information form	“*We want every guideline to have a summary. We actually see this as part of the implementation process. And it will contain patient information.*” – Interviewee 3, guideline developer
**3. Financial**		
Grant provided to support (research aimed at effective) implementation initiatives	Governmental agencies and health insurers provide grants to support (research aimed at effective) implementation initiatives	“*… But scaling up to a broader, local, regional, or national level really requires a bit more, and in these implementation projects, higher budgets or subsidies can be requested.*” – Interviewee 14, governmental agency
Change in reimbursement	Promote implementation by changing care reimbursement	“*Yeah, we don’t take on the role of the executer, but we do try to encourage certain doing by removing the financial incentive.*” – Interviewee 25, health insurer
Incentive applicable and available to the institution	Health insurers offer additional labour compensation to healthcare facilities for creating improvement plans	“*You will receive some additional funding from the health insurers. However, you must, at the very least, create an improvement plan each year.*” – Interviewee 1, national organization
**4. Regulatory**		
Change in legislation or regulation	General Healthcare Agreements between guideline organizations mandate healthcare facilities to implement a certain percentage of the recommendations listed on the national implementation agenda	“*… So of the topics listed on the implementation agenda since the start of the program, 80% must be implemented by the end of this year, and all the current periodic topics must be implemented within two years.*” – Interviewee 1, national organization
Change in licensing, credentialing or accreditation	Assign licensing, credentialing or accreditation to healthcare facilities or professionals that meet certain guideline criteria	“*So it’s a quality document or guideline, or however you want to refer to it, and the Quality Committee checks it. We also make it public on our websites that healthcare facilities comply to it.*” – Interviewee 33, guideline developer
***5. Central***		
*Ask guideline committee for an implementation plan*	Guideline organizations ask guideline committees for an implementation plan	“*What we can do, for instance, in the assessment framework, is include a criterion: is there an implementation or a maintenance plan? So, we explicitly ask them in advance to consider what steps they will take to ensure that this will actually be used in practice.*” – Interviewee 24, governmental agency
*Provide education/support about implementation*	Training implementation science practitioners, provide implementation coaching, provide network for implementation science practitioners	“*And the training from fellows to those experts, promoting the infrastructure, that’s truly the strategy we’re currently focusing on.*” – Interviewee 14, governmental agency
*Share best implementation practices*	Guideline organizations share examples of successful implementation/quality initiatives with guideline organizations and/or healthcare facilities	“*During such meetings, you often try to share good examples of ongoing initiatives that can serve as inspiration for those stakeholders.*” – Interviewee 13, governmental agency
*Collaborative implementation partnerships*	Facilitate agreements and collaborative implementation partnerships between guideline organizations and/or healthcare facilities	“*[Scientific organization] has established [program], where they, together with GPs, encourage collaboration among regions, GPs, and hospitals on several conditions.*” – Interviewee 33, guideline developer
*National implementation agenda*	Guideline organization releases national implementation agenda [47] containing guideline recommendations that healthcare facilities are obliged to implement	“*…Then we attempt to compile the guideline recommendations that have a solid scientific basis. We do this in what’s called the implementation agenda. It’s a central list of recommendations that all healthcare facilities in the Netherlands work on, where we also make agreements with health insurers.*” – Interviewee 1, national organization
*Quality discussions about guideline implementation*	Health insurers conduct quality discussions with healthcare facilities about the implementation of certain guidelines	“*We engage in annual quality discussions with healthcare facilities, in which we assess how these facilities perform based on certain public quality information. If facilities diverge, we always ask: ‘why are you deviating?*’” – interviewee 28, health insurer
*Request improvement plan*	Health insurers ask healthcare facilities for improvement plan	“*On the implementation agenda there are numerous recommendations, and hospitals are required to create a plan demonstrating their commitment to implementing those recommendations. We also monitor those plans. They need to submit these plans to their primary health insurer, and the hospital’s primary health insurer must then approve those plans.*” – interviewee 28, health insurer
*Online collaboration platform*	Online collaboration and knowledge-sharing platform for healthcare facilities, health insurers and policy advisors	“*We have developed a collaboration platform…*” – Interviewee 1, national organization
*Peer-learning sessions for healthcare facilities*	Guideline organization organizes peer-learning sessions for distant healthcare facility quality employees	“*… So we aim to stimulate knowledge exchange. We do this partly through peer-learning sessions and partly through a monthly catch-up session involving all hospitals.*” – Interviewee 1, national organization

^a^Guideline organizations did not address dissemination and implementation strategies within the organizational domain or financial domain involving patients of the modified taxonomy of Mazza et al. [36]. Therefore, these categories were excluded from the Table

^b^Implementation strategies that did not fit in the modified taxonomy of Mazza et al. [36] were classified and added in *italicized *text.

As previously mentioned, strategies were primarily employed by guideline developers. They utilized multiple strategies, often focusing on dissemination. Common dissemination strategies included (1) distributing draft guidelines to their members (healthcare professionals) and other guideline organizations for feedback in the pre-publication commentary phase, (2) advertising guideline materials (e.g. via guideline databases, journals, newsletters, websites, (social) media and patient websites), (3) presenting guideline materials (e.g. conferences and annual meetings) and (4) providing additional implementation supporting materials (e.g. summaries, infographics and pocket cards). A more active implementation strategy that was often employed by guideline developers was educating healthcare professionals about the guideline (e.g. through workshops).

Besides guideline developers, most guideline organizations primarily disseminated guideline materials passively (e.g. via email to end users) and/or worked on the prerequisites for implementation (e.g. providing implementation funding). A few indicated infrequent use of dissemination or implementation strategies, attributing this to limited capacity that required prioritizing tasks. Others felt their efforts did not add value or misaligned with their formal role:

“*…Where in the implementation process should [governmental agency] have a role? Because when it comes to ensuring implementation, I think we’re positioned at the back, dealing with those who lag behind [healthcare facilities and professionals]. So [healthcare facilities and professionals] need to have the time to fall behind first. … In that phase of implementation, I believe we [governmental agency] simply have no role initially.*” – Interviewee 35, governmental agency.

There were also examples of organizations that were more actively involved. For example, one national organization created a national implementation agenda containing well-substantiated guideline recommendations that healthcare facilities were obliged to implement. Furthermore, they aided healthcare facilities by sharing benchmark information, recruiting champions, offering a knowledge-sharing platform and organizing peer-learning sessions. Another example is that health insurers engaged in discussions with healthcare facilities regarding benchmark information indicating implementation of specific guidelines. If implementation fell short of the standards, they requested healthcare facilities to formulate and execute improvement plans. Moreover, they indicated promoting implementation by adjusting financial reimbursements for guideline-compliant care.

### Process, outcome and impact monitoring and evaluation

Guideline organizations utilized diverse monitoring and evaluation methods regarding the implementation of guidelines (Table 4). They primarily focused on process and outcome assessments, measuring guideline use and changes in healthcare professionals and facilities’ practice. Guideline developers mainly referred to clinical peer review visits as their primary and often only formal evaluation method. These visits to care departments involved external healthcare professionals who assessed the care processes, including the implementation of guidelines, and provided improvement recommendations.

**Table 4:** Implementation process, outcome and impact monitoring and evaluation methods employed by the guideline organizations

Implementation process, outcome and impact monitoring and evaluation methods	Exemplary quotes
***1.*** **Implementation process monitoring and evaluation**	
Staff of guideline developer holds meetings to evaluate and discuss how implementation process improvements can be made	“*Well, in staff meetings, it’s discussed how things went and how they can be improved. Especially focusing on how they can be improved.*” – Interviewee 19, guideline developer
Reviewing user numbers of guideline materials (e.g. website visitors)	“*… And then it was like: we’re providing cases, but is anyone actually looking at them? So, the communications advisor inquired: how frequently are they clicked on? And then we knew, okay, we’re doing it for a reason because it’s being read.*” – Interviewee 15, guideline developer
Surveying use of guideline during members’ meeting	“*Yes, we once used Mentimeter to measure the awareness of the [guidelines] and the guideline database. However, it appears that very few people are familiar with the guideline database and, in practice, hardly use it at all.*” – Interviewee 33, guideline developer
Gathering information on guideline utilization informally (calls, emails, conversations, focus groups)	“*We simply asked. Actually, we called various hospitals and inquired.*” – Interviewee 34, guideline developer
Study exploring the utilization of guidelines in practice (e.g. through interviews with healthcare professionals)	“*Last year, we conducted a study on the utilization of guidelines in practice. … We interviewed 60 teams and assessed the extent to which they still used guidelines.*” – Interviewee 29, guideline developer
**2. Implementation outcomes monitoring and evaluation**	
Governmental agencies evaluate quality data (e.g. ‘Transparency Calendar’) and provide feedback to healthcare facilities on risk detection (audit and feedback)	“*Because we look at the Transparency Calendar and all available benchmark information, posing targeted questions based on that. That feedback on risk detection leads to improvement.*” – Interviewee 35, governmental agency
National organization develops audit and feedback based on self-reported data from healthcare facilities and health insurer claims data	“*All healthcare facilities report their progress on these subjects. Thus, we can track how each hospital is performing regarding the implementation of these subjects. So, our central role is monitoring and intervening when we notice certain subjects lagging behind the intended pace.*” – Interviewee 1, national organization
Guideline developer develops audit and feedback	“*So, then we reluctantly started making audit and feedback ourselves. For certain aspects of care, we conducted practice variation studies and repeated them to see what had changed.*” – Interviewee 32, guideline developer
Evaluation through clinical peer review	“*During the clinical peer review visit, various norms and themes arise, but the implementation of guidelines and new knowledge is a topic that is indeed discussed during these visits. Yes.*” – Interviewee 22, guideline developer
**3. Impact monitoring and evaluation**	
The Court of Audit evaluated both the potential and actual savings achieved by the Appropriate Care program	“*… However the Court of Audit has also evaluated this, and they have actually said, ‘You produce very nice, well-substantiated reports, but ultimately, not much happens with those recommendations’.*” – Interviewee 23, governmental agency
**4. Combination of implementation process, outcomes and/or impact monitoring**	
‘Appropriate Care Program’: governmental agency (1) compares existing guidelines and outcome data (e.g. health insurer claims data and GP data), (2) establishes improvement commitments, (3) monitors and evaluates outcome data, implementation activities of collaborating guideline organizations and their own and (4) sends monitor reports to guideline developers and Ministries of Public Health, Welfare and Sport (VWS)	“*We do engage in annual monitoring: identifying which improvement commitments have been realized and which haven’t. This focuses more on a proximal level, in which our stakeholders, for instance, raise certain topics or modify guidelines.*” – Interviewee 6, governmental agency
Governmental agency collects outcomes and impact of the guideline projects that they funded (including cost-effectiveness research) and provides reports to the Ministries of Public Health, Welfare and Sport (VWS)	“*We can track these kinds of effects from [governmental agency] as well. We also demonstrate them to the Ministry of Public Health, Welfare, and Sport (VWS). ‘Look, the research we initially encouraged is indeed yielding the outcomes you desire.’ That’s about demonstrating the impact.*” – Interviewee 14, governmental agency
Health insurer monitors and evaluates through examining claims data (benchmark information), information from insured persons and assessing how healthcare facilities execute their improvement plans	“*In those quality discussions you’re reviewing those indicators. And then you ask for an explanation. And if that explanation is insufficient, you request an improvement plan and then you either check again after six months or a year. A year later, you’ll have access to that quality information again. … And you simply ask the hospital: show us what you’ve done. Show us how you’ve improved.*” – Interviewee 28, health insurer

Besides clinical peer review, most guideline developers did not conduct additional formal monitoring and evaluation activities. Some occasionally measured guideline usage informally through member or healthcare facilities inquiries or by tracking website/document users. A few also developed audit and feedback indicators for members to use or commissioned external researchers to evaluate guideline utilization.

Besides guideline developers, a few other guideline organizations employed formal recurrent monitoring and evaluation methods. As previously stated, one national organization developed benchmark information using routinely collected health insurer claims data and self-reported data from healthcare facilities, to assess whether recommendations had been implemented. Health insurers also utilized the benchmark information and conducted additional evaluations based on their health insurer claims data. They discussed their analyses with healthcare facilities and requested improvement plans accordingly. Additionally, with the aid of several other guideline organizations and data from healthcare facilities, one governmental agency developed benchmark information based on certain guidelines (‘Transparency Calendar’). Healthcare facilities could use this benchmark information to implement targeted improvements, and governmental agencies used the data for regulation.

Another evaluation method used by a governmental agency was the ‘Appropriate Care’ program, which compared recommendations from existing guidelines with available scientific evidence and outcome data. Improvement commitments were then collaboratively developed with relevant guideline organizations, with the goal of enabling both guideline organizations and end users to enhance their guidelines, implementation strategies and clinical practice. Subsequent changes in outcome data were then monitored and reported.

A last evaluation method was conducted by another governmental agency, which collected outcome and impact data from the (local) guideline projects that they funded and reported this information to the Ministries of Public Health, Welfare and Sport (VWS).

Guideline organizations reported encountering several challenges in monitoring and evaluation. A commonly cited issue was the infrequency of assessments, such as those conducted through clinical peer reviews:

“*The downside of that instrument, in my opinion, is that it takes 5 years before all departments have been visited. … So it’s not a very strong tool for implementing all guidelines, but it is a robust means to prompt departments to be aware of guidelines and their implementation.*” – Interviewee 32, guideline developer.

Determining the desired level of implementation was also perceived as difficult. Additionally, it was not always clear for guideline organizations whether the impracticality of the guidelines or the need for increased dissemination and implementation efforts was the issue. Another frequently mentioned challenge was the absence of a dedicated budget for monitoring and evaluation. Moreover, distinguishing whether improvements in care resulted from active guideline implementation or external factors – such as global initiatives, increased public awareness or heightened media attention – remained difficult.

## Discussion

Amidst growing healthcare pressures from rising expenditures, population ageing, constrained staffing and environmental concerns, guidelines can be crucial tools for steering practices towards appropriate care. Yet their impact hinges on implementation in practice. This study explored how Dutch guideline organizations plan, execute, monitor and evaluate their implementation efforts.

This qualitative study reveals that guideline organizations recognize and endorse the significance of implementation. Nevertheless, this recognition does not consistently translate into tailored implementation actions. In terms of implementation planning, opportunities exist for guideline organizations to tailor implementation strategies by pre-identifying barriers, engaging stakeholders and employing implementation TMFs – approaches known to enhance guideline uptake and impact [24, 25, 34, 35, 48]. However, these opportunities, such as developing the implementation plan based on identified barriers specific to the guideline or providing input on the applicability of guidelines during the commentary round, are not always fully utilized. Even when employed, the subsequent step of selecting and tailoring implementation strategies based on the identified barriers, stakeholder insights or implementation TMF guidance is frequently neglected. This suggests that the ability to adapt guidelines and implementation efforts to different clinical and organizational contexts is not yet embedded in guideline organizations’ processes [22, 49, 50].

This issue is not unique to the Netherlands. A recent review by the current authors [27], which included studies on guideline implementation in hospital care from countries such as the USA, Australia and Canada reported similar findings across guideline organizations worldwide. However, a separate review by Peters et al. [35], which looked at both implementation efforts of guideline organizations and local initiatives, found that efforts to select and tailor guideline implementation strategies were more informed by implementation planning approaches compared to their earlier 2015 review [37]. This positive shift may be due to increased awareness of the expanding research on optimizing implementation efforts [48].

Limited resources (time and funding) and a lack of implementation knowledge or motivation within the guideline organizations and guideline committees hinder effective selection and tailoring of implementation strategies. These barriers also mirror resource, knowledge and motivational issues reported in previous studies on guideline organizations in other countries [22, 50]. For instance, Gagliardi’s study on implementation approaches of international guideline developers found that most lacked funding for implementation. Even those organizations with a mandate to promote use of their guidelines lacked dedicated financial support [22]. This points to the need for stronger institutional support, resources and education around implementation processes to bridge the gap between guideline development and practical application.

We also found that Dutch guideline organizations employ a large variety of primarily dissemination strategies. Guideline developers predominantly aim their strategies at healthcare professionals, whereas other organizations (e.g. health insurers and government agencies) mainly target facilities. Guideline developers and patient organizations extend their strategies to patients. Commonly used strategies included distributing, advertising and presenting guideline materials, along with providing additional implementation supporting materials. These strategies are commonly found in other guideline implementation studies, reinforcing their widespread use [22, 35, 49, 51]. The reliance on these ‘basic’ dissemination strategies points to an underutilization of more interactive (e.g. training, audit and feedback) or tailored implementation strategies, which are generally more effective in promoting guideline adherence [4, 26]. Additionally, there is significant room for improvement in making Dutch guidelines more practical for clinical use. Weller et al. found that while Dutch medical specialists consider guidelines to be one of the most valuable information sources to update their knowledge, they are not used as the primary reference for medical decision-making during consultations [52]. Currently, it is not possible to quickly and accurately search them based on specific questions or patient characteristics [53]. Guideline organizations could improve usability by enhancing the search functionality of the central guideline and encouraging software providers to integrate guidelines into electronic patient records. Both strategies could potentially be further strengthened by incorporating artificial intelligence to generate guideline-based clinical recommendations [54]. In this way, the clinical applicability of guidelines is enhanced to better support medical decision-making.

We expanded the Mazza et al. taxonomy [36, 37], as not all implementation strategies that we found in our study fit the predefined strategies. We also added a new, central domain to the existing five. The scope and impact of guideline organizations differ from those of locally driven, healthcare facility-based implementation initiatives. As guideline organizations work to improve national guideline adherence, they often require broader, system-level strategies. The original taxonomy may be better suited for local, internal healthcare facility-focused initiatives rather than our nationally driven ones.

With respect to monitoring and evaluation methods, only a few guideline organizations assess the process, outcome or impact of guideline implementation. Guideline developers rely on informal methods (e.g. discussions with members) for process evaluation and clinical peer review or occasional one-time studies for outcome evaluation. The lack of regular and structured monitoring and evaluation often leave them uncertain about the effectiveness of their implementation efforts in supporting end users and improving patient care.

Other guideline organizations (e.g. health insurers and governmental agencies) predominantly rely on benchmark information, primarily medical claims data from health insurers, for outcome and impact evaluation and audit and feedback. Audit and feedback generally yield small but potentially important improvements in practice [55, 56]. Furthermore, medical claims data are readily available and, therefore, reduces the administrative burden and cost of evaluations. However, using medical claims data for evaluation also comes with its limitations. The data are primarily collected for purposes other than outcome and impact evaluation and, therefore, may be less suitable [57]. Moreover, de Weerdt et al. found that healthcare professionals accept medical claims data only for certain clinical topics and under certain conditions [58]. Therefore, it should be used with caution.

Looking at the overall guideline process, it appears that greater emphasis is placed on developing guidelines rather than implementing them. While a few guideline organizations take a more systematic approach to implementation, exemplified by the establishment of dedicated implementation teams, these are exceptional cases. Generally, there seems to be a lack of a detailed, structured, mutually aligned step-by-step plan for guideline organizations regarding implementation. This is in contrast to the systematic and coordinated approach employed in guideline development [29]. Although a diverse range of different dissemination and implementation strategies is employed, implementation is often seen as an afterthought, not carefully considered early in the development process. For some guideline organizations, it appears more as a formality than a fully integrated step to facilitate practice change. As a result, the potential impact of guidelines may not be fully realized.

We have identified both implications for follow-up research and practice. Further research into similarities and differences in how guideline organizations in other countries approach implementation, their allocation of roles and best practices can yield valuable lessons for optimizing implementation efforts. Furthermore, enhancing current implementation practices requires the allocation of dedicated time, resources and knowledge support. This enables the systematic completion of the entire cycle encompassing guideline implementation planning (which prioritizes pre-identifying barriers, engaging stakeholders and utilizing implementation TMFs), as well as execution, monitoring and evaluation. Guideline organizations could, hereby, focus efforts on specific guideline recommendations backed by substantial evidence and with significant impact (e.g., affected patient numbers, illness severity, resource savings or environmental considerations). We believe it would be helpful if this choice of impactful topics would be made in a process of national implementation agenda-setting, to be able to align stakeholders’ efforts and to embed guideline implementation in broader policies aimed at improving quality and efficiency of care.

Furthermore, we have identified several suggestions for improvement of the AQUA guideline. The envisioned roles and responsibilities of each guideline organization (and local stakeholder) can be made more explicit. Moreover, the guideline does reference a useful implementation theory-based checklist for pre-identifying barriers. However, it lacks practical guidance on subsequent steps after barriers have been identified. For example, it does not provide direction on how to develop an effective implementation plan, utilize tools to select appropriate implementation strategies (e.g. the CFIR-ERIC matching tool [59]), engage stakeholders in tailoring these strategies or where to find useful implementation TMFs. It would also be beneficial to gather best implementation practices from guideline organizations and compile an overview of existing national, regional and local structures, organizations and initiatives for collaboration. This information could be included in an additional practical implementation manual, allowing guideline organizations to avoid reinventing the wheel. Nationwide guideline development and implementation involves extensive programs and significant resources. Making this process more efficient and effective can yield a substantial positive impact.

### Strengths and limitations

A major strength of this study is its comprehensive approach, encompassing critical aspects of the implementation cycle, from planning to evaluation. This approach provides a thorough overview of implementation mechanisms, avoiding isolation of specific aspects. It offers a broad understanding of the variety of implementation planning, execution and monitoring and evaluation efforts undertaken by guideline organizations. However, to better direct improvement initiatives where they are most needed, quantitative data on the frequency of specific approaches would be useful, requiring additional quantitative research.

Another strength lies in the diverse range of participating guideline organizations. This diversity ensures representation of varied roles, perspectives, professions and specializations. However, it is important to note that the representatives participating in the study may have had a higher affinity for implementation, potentially leading to selection bias. We were not able to determine the exact impact this may have had on the results. One possibility is that guideline organizations already actively engaged in implementation initiatives may have been more inclined to respond to the interview invitation. On the other hand, organizations that still had significant progress to make in implementation and faced implementation challenges may have also been motivated to participate. This could have resulted in either an overestimation or underestimation of nationwide implementation efforts.

While thematic saturation was achieved, the interviewed organizations may not fully represent the entire spectrum of guideline organizations and their guideline implementation practices.

Lastly, the study benefited from the application of theories related to implementation planning, execution, monitoring and evaluation, as well as the validated taxonomy of Mazza et al. [36], to guide data collection and/or analysis.

## Conclusions

Guideline organizations play a crucial role in facilitating the implementation of guidelines in practice. This study revealed that although Dutch guideline organizations acknowledged and endorsed the importance of implementation, implementation efforts often appeared unstructured, treated as a mere formality and an afterthought. Dissemination and implementation strategies were minimally selected and tailored based on implementation planning approaches that have previously demonstrated to optimize implementation efforts. Primarily dissemination and occasionally implementation strategies were used to improve guideline uptake and impact. Additionally, the lack of regular, structured monitoring and evaluation left uncertainties about the effectiveness of implementation efforts in supporting guideline end users and improving health outcomes. Suggested follow-up research and practice enhancements could strengthen central-level guideline implementation efforts, forming a basis for more effective implementation locally, ultimately benefiting patient care.

## Supplementary Information

Additional file 1. Completed COREQ checklist

Additional file 2. Interview guide

Additional file 3. Coding tree

## Data Availability

Interview data that were used and analysed during the current study are available from the corresponding author on reasonable request.

## Abbreviations

COREQ: Consolidated criteria for reporting qualitative research
TMFs: Theories, models and frameworks
