# Supplementary material for: The role of guideline organizations in nationwide guideline implementation: a qualitative study
Source: Health Res Policy Syst. 2024 Dec 23;22:174. doi: 10.1186/s12961-024-01253-0 (PMC11668013; doi:10.1186/s12961-024-01253-0)
Supplement: Supplementary file 1 — Additional file 1. Completed COREQ checklist [file 12961_2024_1253_MOESM1_ESM.docx]

Appendix 1. Completed COREQ (COnsolidated criteria for REporting Qualitative research) Checklist

| **Topic** | **Guide Questions/description** | **Details (manuscript page number, if reported)** |
| --- | --- | --- |
| Domain 1: Research team and reflexivity | | |
| Personal characteristics | | |
| 1. Interviewer/ facilitator | Which author(s) conducted the interview or focus group? | Interviews were conducted by one or two researchers:  AT, TB, AG. (p. 7) |
| 2. Credentials | What were the researcher’s credentials? (e.g. PhD, MD) | AT: MSc.  HM: PhD  TB: BSc, received MSc after completing the internship that this study was part of.  AG: BSc, received MSc after completing the internship that this study was part of.  IvB: PhD  DD: Prof.Dr.  MdB: Prof.Dr. |
| 3. Occupation | What was their occupation at the time of the study? | AT: PhD researcher  HM: assistant professor  TB: MSc student/intern  AG: MSc student/intern  IvB: advisor and researcher  DD: professor and chief scientific officer  MdB: professor and director |
| 4. Gender | Was the researcher male or female? | Female: AT, HM, AG, IvB, DD, MdB  Male: TB |
| 5. Experience and training | What experience or training did the researcher have? | The interviewers (AT, TB, AG) had a background in health policy studies, were trained in interviewing techniques and had interview experience. (p. 8) |
| Relationship with participants | | |
| 6. Relationship established | Was a relationship established prior to study commencement? | No prior relationship was established between the researchers and participants |
| 7. Participant knowledge of the interviewer | What did the participants know about the researcher? (e.g. personal goals, reasons for doing the research) | Participants knew the occupations of the interviewers, where they worked and the purpose of the research. |
| 8. Interviewer characteristics | What characteristics were reported about the interviewer/facilitator? (e.g. bias, assumptions, reasons and interests in the research topic) | DD and IvB, besides their roles as researchers, are employed by Zorginstituut Nederland, a guideline organization. While this may introduce potential bias or conflicts of interest, we have mitigated this by extensively addressing and discussing the matter in our group discussions on study design and result interpretation. (p. 32) |
| Domain 2: study design | | |
| Theoretical framework | | |
| 9. Methodological orientation and theory | What methodological orientation was stated to underpin the study? (e.g. grounded theory, discourse analysis, ethnography, phenomenology, content analysis) | The study was underpinned by 1) a review of Peters et al. (2022), 2) the ‘taxonomy of strategies for achieving guideline implementation and compliance’ of Mazza et al. (2013), expanded by Gagliardi & Alhabib (2015) and 3) the principles of logic models. (p. 8-11) |
| Participant selection | | |
| 10. Sampling | How were participants selected? (e.g. purposive, convenience, consecutive, snowball) | Guideline organizations were eligible if they were scientific/professional organizations, knowledge institutes, governmental agencies, health insurers or other national (umbrella) organizations that developed guidelines, published them and/or actively supported their use in Dutch clinical practice. We recruited representatives from guideline organizations based on their insights into their organization's role or direct involvement in implementing guidelines. We used purposive sampling methods to recruit a broad sample of these representatives. Furthermore, representatives who were interviewed were asked if they knew additional representatives who could participate (snowball sampling). (p. 7) |
| 11. Method of approach | How were participants approached? (e.g. face to face, telephone, mail, e-mail) | Participants were contacted via email or telephone. (p. 7) |
| 12. Sample size | How many participants were in the study? | A total of 35 participants from 24 different guideline organizations were interviewed. (p. 13) |
| 13. Non-participation | How many people refused to participate or dropped out? Reasons? | Numbers of refusals were not recorded. |
| Setting | | |
| 14. Setting of data collection | Where was the data collected? (e.g. home, clinic, workplace) | Interviews were held via videoconference or in-person (e.g. at their workplace) based on participant preference. (p. 7) |
| 15. Presence of non-participants | Was anyone else present besides the participants and researchers? | Only the researchers and participants were present. |
| 16. Description of sample | What are the important characteristics of the sample? (e.g. demographic data, date) | Occupation(s) and role in relation to guideline implementation. (p. 13) |
| Data collection | | |
| 17. Interview guide | Were questions, prompts, guides provided by the authors? Was it pilot tested? | Interview topic guide with prompts (Appendix 2) was developed and used during the interviews. The topic guide was not pilot tested, but it was extensively reviewed within the research team. (p. 8 and Appendix 2) |
| 18. Repeat interviews | Were repeat interviews carried out? If yes, how many? | No repeat interviews were required. |
| 19. Audio/visual recording | Did the research use audio or visual recording to collect the data? | Interviews were audio-recorded. (p. 11) |
| 20. Field notes | Were field notes made during and/or after the interview or focus group? | Field notes were made during the interview. |
| 21. Duration | What was the duration of the interviews or focus group? | Interviews ranged from 30-100 minutes. (p. 8) |
| 22. Data saturation | Was data saturation discussed? | Data collection continued until no new themes emerged, signifying data saturation. (p. 8) |
| 23. Transcripts returned | Were transcripts returned to participants for comment and/or correction? | Participants received an interview summary for commenting (member checking). (p. 8) |
| Domain 3: analysis and findings | | |
| Data analysis | | |
| 24. Number of data coders | How many data coders coded the data? | Initially, two researchers (AT and either TB or AG) independently coded the first eight interviews to align coding. Subsequently, one researcher (TB, AG, or AT) coded the rest, cross-checked by a second researcher (AT or HM). (p. 11) |
| 25. Description of the coding tree | Did authors provide a description of the coding tree? | The final coding tree is provided in Appendix 3. (p. 11 and Appendix 3) |
| 26. Derivation of themes | Were themes identified in advance or derived from the data? | Both deductive and inductive coding were used. For deductive coding, the researchers developed an initial codebook, based on the aforementioned theoretical constructs. In addition, open coding was used to include interesting themes that emerged from the data. Throughout the coding process, the codebook was updated iteratively. (p. 11) |
| 27. Software | What software, if applicable, was used to manage the data? | Coding was conducted using MAXQDA (version 2022). (p. 11) |
| 28. Participant checking | Did participants provide feedback on the findings? | Participants did not provide feedback on the findings. |
| Reporting | | |
| 29. Quotations presented | Were participant quotations presented to illustrate the themes/findings? Was each quotation identified? (e.g. participant number) | Participant quotes were presented to illustrate te findings throughout the results section. Each quote is identified through a participant number and his/her corresponding type of guideline organization. (p. 14-24) |
| 30. Data and findings consistent | Was there consistency between the data presented and the findings? | We strived to present the study findings with clarity and consistency, aiming to accurately reflect the collected data. |
| 31. Clarity of major themes | Were major themes clearly presented in the findings? | Yes, major themes are clearly presented throughout the results section, both in text and in tables. (p. 14-24) |
| 32. Clarity of minor themes | Is there a description of diverse cases or discussion of minor themes? | Yes, diverse cases and minor themes are clearly presented throughout the results section, both in text and in tables. (p. 14-24) |
