# Supplementary material for: The role of guideline organizations in nationwide guideline implementation: a qualitative study
Source: Health Res Policy Syst. 2024 Dec 23;22:174. doi: 10.1186/s12961-024-01253-0 (PMC11668013; doi:10.1186/s12961-024-01253-0)
Supplement: Supplementary file 2 — Additional file 2. Interview guide [file 12961_2024_1253_MOESM2_ESM.docx]

Appendix 2. Interview guide

Translated from Dutch.

## Introductory text

First of all, Thank you for participating in this study. **introduce yourself and your occupation**. The subject of this research revolves around gaining insight into the processes followed by guideline organizations in the dissemination and/or implementation of guidelines. The key topics within this research include dissemination and implementation strategies and the factors that can influence the implementation process. Topics such as implementation planning and evaluation are also explored. To provide a comprehensive overview of the different roles, perspectives and experiences, representatives from various guideline organizations involved in the development, implementation planning, dissemination, implementation and/or evaluation of guidelines have been invited to participate in this research.

If you participate in this interview, you agree to the use of your answers for this study. Your data and responses will be treated confidentially and will not be shared with third parties. Throughout the transcription and analysis of the interview, responses will be handled anonymously. The interview will be recorded using an audio recorder, and the audio file will be deleted after it has been transcribed verbatim, with names of individuals anonymized. These transcripts will be stored in a secure database for a period of 15 years. After completing the transcript, I will send a summary of the interview so that you can provide any additional comments.

Do you have any questions before we begin?

Are you okay with me starting the recording? **Start the recording**

You have read the information form, and if you had any questions, they have been answered by the researcher. The consent form has been signed by both of us. Can you confirm this for me?

## Introduction

We will start this interview with some general questions about your work within your organization.

| *Topic* | *Questions/constructs* |
| --- | --- |
| Introduction | Can you tell me about your role at [organization]?  Can you provide an overview of your organization’s role in the process of developing, disseminating, and implementing guidelines?   - *Example* - *In which guidelines have you recently been actively engaged?* |

## Planning phase

The following questions are about elements of the implementation planning phase.

| *Topic* | *Questions/constructs* |
| --- | --- |
| General | How are decisions made regarding the selection of specific dissemination and implementation strategies? |
| Tailoring interventions | Are methods employed to tailor dissemination and implementation strategies for better uptake in practice?   - *Who is responsible for this within the organization?* |
| Engaging stakeholders | With which organizations do you collaborate in the process of guideline development, dissemination and/or implementation?  Who are the guideline end-users?  How are they/their perspectives on the guideline and implementation identified?  Are end-users actively involved in planning dissemination and implementation strategies? Are dissemination and implementation strategies actually tailored to the needs of the end-users?   - *How?* |
| Pre-identified barriers and facilitators | Do you identify potential implementation barriers or facilitators prior to tailoring the dissemination and implementation strategies?   - *How are they identified and taken into account?* - *Examples from recent guidelines* |
| Using theories/ models/ frameworks | Does your organization make use of theories, models or frameworks to guide implementation? Which ones?  If not/unclear, has the organization developed guidelines/manuals for planning dissemination and implementation strategies?   - *To what extent do they provide support?* - *Are the guidelines/manuals actually utilized?* - *Could we receive these guidelines/manuals?* |

**Implementation phase**The following questions are about the actual dissemination and implementation of guidelines.

| *Topic* | *Questions/constructs* |
| --- | --- |
| Dissemination and implementation strategies | Which strategies for the dissemination of guidelines are utilized by your organization?  Which strategies for implementation of guidelines are utilized by your organization?   - *Examples of strategies* - *Experiences with strategies* - *Resources utilized and available for implementation*   **Examples of strategies**  Professional: education, audit and feedback, reminders, clinical peer review, publications, media  Financial: change in reimbursement, implementation grants, fines  Organizational: integration of services, healthcare professional satisfaction, changes in the medical records system, changes in the healthcare setting  Regulatory: change in regulation/legislation, change in licensing, credentialing or accreditation  Patient: education, print material (patient summary) |
| Implementation barriers and facilitators | What barriers have you encountered during the process of guideline implementation?  What facilitators have you encountered during the process of guideline implementation?  To what extent does implementation proceed according to the preconceived plan? |

**Monitoring and evaluation phase**The following questions are about the monitoring and evaluation of the implementation efforts, (process), outcomes and impact.

| *Topic* | *Questions/constructs* |
| --- | --- |
| Process, outcome and impact evaluation | What happens after the implementation activities have been completed?  To what extent is your organization involved in the phase after the implementation activities are completed (monitoring and evaluation phase)?  Is the implementation approach (planning and execution of implementation strategies) evaluated afterwards?   - *In what way?* - *Examples of outcomes of those evaluations*   Are the outcomes and impact of the implementation of the guidelines on healthcare monitored/evaluated?   - *In what way?* - *Which outcome and impact indicators are being considered? (patient outcomes, health outcomes, cost-effectiveness, etc.)* - *Examples of recent outcomes* |

**Closing**Finally, I have some concluding questions.

| *Topic* | *Questions/constructs* |
| --- | --- |
| Guideline | What is your opinion on guidelines as a bridge between science, policy, and healthcare practice?  What do you think about the current efforts in disseminating and implementing guidelines in the Netherlands?   - *How would you assess the efforts of your organization in this regard?* |
| Good and bad examples | Can you provide an example of a guideline where the dissemination and implementation process has been successful?   - *Why was it effective/successful?*   Can you provide an example of a guideline where the dissemination and implementation process has not been/was less successful?   - *Why was it ineffective/unsuccessful?*   How can the dissemination and implementation process of guidelines be improved?   - *Examples?* |
| Closing | Could you recommend other individuals, within your organization or other key entities like governmental organizations, who would be valuable for us to interview?  Is there anything else you would like to add that we may not have covered but is relevant to the discussion?  Do you have any final questions for me? |
