# Supplementary material for: The role of guideline organizations in nationwide guideline implementation: a qualitative study
Source: Health Res Policy Syst. 2024 Dec 23;22:174. doi: 10.1186/s12961-024-01253-0 (PMC11668013; doi:10.1186/s12961-024-01253-0)
Supplement: Supplementary file 3 — Additional file 3. Coding tree [file 12961_2024_1253_MOESM3_ESM.docx]

Appendix 3. Final coding tree

| **Code System** |
| --- |
| Current state of implementing guidelines |
| Value of guidelines as bridge between science, policy & practice |
| Solutions/ideas for implementation problem |
| Roles organizations in implementation process |
| Roles organizations in implementation process\Role/responsibility governmental agencies |
| Roles organizations in implementation process\Role/responsibility NZa |
| Roles organizations in implementation process\Role/responsibility IKNL |
| Roles organizations in implementation process\Role/responsibility ZonMw |
| Roles organizations in implementation process\Role/responsibility NFU |
| Roles organizations in implementation process\Role/responsibility patient federation/patient organizations |
| Roles organizations in implementation process\Role/responsibility ZE&GG |
| Roles organizations in implementation process\Role/responsibility VWS |
| Roles organizations in implementation process\Role/responsibility insurers |
| Roles organizations in implementation process\Role/responsibility ZIN |
| Roles organizations in implementation process\Role/responsibility FMS/KIMS |
| Roles organizations in implementation process\Role/responsibility scientific/professional organizations |
| Roles organizations in implementation process\Role/responsibility IGJ |
| Roles organizations in implementation process\Role/responsibility NVZ |
| Roles organizations in implementation process\Role/responsibility NVVC |
| Roles organizations in implementation process\Role/responsibility healthcare professionals/facilities |
| Roles organizations in implementation process\Role/responsibility NVALT |
| Roles organizations in implementation process\Role/responsibility V&VN |
| Roles organizations in implementation process\Role/responsibility NHG |
| Roles organizations in implementation process\Role/responsibility ZKN |
| Organization has (no) implementation team |
| 1. Implementation planning approach |
| 1. Implementation planning approach\No official implementation phase |
| 1. Implementation planning approach\No structural implementation approach currently, aspiration for one |
| 1. Implementation planning approach\Implementation planning approach not linked to implementation strategies |
| 1. Implementation planning approach\Emphasis on doing instead of planning first |
| 1. Implementation planning approach\Whatever implementation strategies come to mind, unsubstantiated |
| 1. Implementation planning approach\Based on sensitivity topic |
| 1. Implementation planning approach\Based on urgency topic, common problem |
| 1. Implementation planning approach\Based on available implementation grants SKMS |
| 1. Implementation planning approach\Depending on preferences and capacity guideline committee |
| 1. Implementation planning approach\Additional implementation research |
| 1. Implementation planning approach\Conducting a stakeholder analysis |
| 1. Implementation planning approach\Following an implementation course |
| 1. Implementation planning approach\No pre-identification barriers or facilitators |
| 1. Implementation planning approach\During guideline development thinking of implementation |
| 1. Implementation planning approach\Choosing strategies based on easiness and costs |
| 1. Implementation planning approach\Based on successful/impactful strategies of other guideline organizations or similar projects |
| 1. Implementation planning approach\working with marketing agency, educational/implementation expert |
| 1. Implementation planning approach\Collaborating with implementation practice experts |
| 1. Implementation planning approach\Guideline pilot testing |
| 1. Implementation planning approach\Based on guideline characteristics |
| 1. Implementation planning approach\Implementation plan |
| 1. Implementation planning approach\Engaging stakeholders |
| 1. Implementation planning approach\Pre-identifying facilitators |
| 1. Implementation planning approach\Pre-identifying barriers |
| 1. Implementation planning approach\Using theories/models/frameworks |
| 2. Dissemination/implementation methods |
| 2. Dissemination/implementation methods\No implementation strategies |
| 2. Dissemination/implementation methods\Central domain |
| 2. Dissemination/implementation methods\Central domain\Collaboration platform for distant healthcare facilities, insurers, policy advisors |
| 2. Dissemination/implementation methods\Central domain\Request implementation plan (for approval Register) |
| 2. Dissemination/implementation methods\Central domain\Provide education/support about implementation |
| 2. Dissemination/implementation methods\Central domain\Share best implementation practices with guideline organizations + healthcare facilities |
| 2. Dissemination/implementation methods\Central domain\Collaborative implementation partnerships |
| 2. Dissemination/implementation methods\Central domain\National implementation agenda |
| 2. Dissemination/implementation methods\Central domain\Quality discussions insurers and healthcare facilities |
| 2. Dissemination/implementation methods\Central domain\Peer-learning sessions |
| 2. Dissemination/implementation methods\Central domain\Request healthcare facilities to submit improvement plan |
| 2. Dissemination/implementation methods\Regulatory domain |
| 2. Dissemination/implementation methods\Regulatory domain\Change legislation or regulation |
| 2. Dissemination/implementation methods\Regulatory domain\Change in licensing, credentialing or accreditation |
| 2. Dissemination/implementation methods\Regulatory domain\Change in licensing, credentialing or accreditation\guideline organization publishes white list of healthcare facilities that have proven to meet the quality criteria |
| 2. Dissemination/implementation methods\Regulatory domain\Change in licensing, credentialing or accreditation\guideline developer manages network of recognized quality consultants |
| 2. Dissemination/implementation methods\Regulatory domain\Change in licensing, credentialing or accreditation\guideline developer manages network of peripheral accreditation employees |
| 2. Dissemination/implementation methods\Organizational domain |
| 2. Dissemination/implementation methods\Financial domain |
| 2. Dissemination/implementation methods\Financial domain\Provide budget for implementation research/project |
| 2. Dissemination/implementation methods\Financial domain\Change in reimbursement |
| 2. Dissemination/implementation methods\Financial domain\Incentive (group or institutional financial reward or benefit) |
| 2. Dissemination/implementation methods\Patient domain |
| 2. Dissemination/implementation methods\Patient domain\Advertise guideline materials to patients |
| 2. Dissemination/implementation methods\Patient domain\Advertise guideline materials to patients\Patient organization website publication |
| 2. Dissemination/implementation methods\Patient domain\Advertise guideline materials to patients\Patient version of guideline/patient information form |
| 2. Dissemination/implementation methods\Patient domain\Advertise guideline materials to patients\Patient website publication |
| 2. Dissemination/implementation methods\Professional domain |
| 2. Dissemination/implementation methods\Professional domain\Distribute guideline materials |
| 2. Dissemination/implementation methods\Professional domain\Distribute guideline materials\Issue draft guideline for commenting |
| 2. Dissemination/implementation methods\Professional domain\Distribute guideline materials\Mass mailing (electronic) final guideline |
| 2. Dissemination/implementation methods\Professional domain\Provide feedback on guideline compliance and information |
| 2. Dissemination/implementation methods\Professional domain\Enable self-audit |
| 2. Dissemination/implementation methods\Professional domain\Present guideline materials at meetings |
| 2. Dissemination/implementation methods\Professional domain\Present guideline materials at meetings\Conference presentation |
| 2. Dissemination/implementation methods\Professional domain\Clinical peer review |
| 2. Dissemination/implementation methods\Professional domain\Recruit champions |
| 2. Dissemination/implementation methods\Professional domain\Educate groups about guideline intent/benefits |
| 2. Dissemination/implementation methods\Professional domain\Educate groups about guideline intent/benefits\Workshop |
| 2. Dissemination/implementation methods\Professional domain\Educate groups about guideline intent/benefits\(Online) education material |
| 2. Dissemination/implementation methods\Professional domain\Educate groups about guideline intent/benefits\Webinar |
| 2. Dissemination/implementation methods\Professional domain\Educate groups about guideline intent/benefits\Micro learning quiz |
| 2. Dissemination/implementation methods\Professional domain\Educate groups about guideline intent/benefits\Instruction video's |
| 2. Dissemination/implementation methods\Professional domain\Educate groups about guideline intent/benefits\E-learnings |
| 2. Dissemination/implementation methods\Professional domain\Advertise guideline materials |
| 2. Dissemination/implementation methods\Professional domain\Advertise guideline materials\Publish (in) book |
| 2. Dissemination/implementation methods\Professional domain\Advertise guideline materials\Animated short film |
| 2. Dissemination/implementation methods\Professional domain\Advertise guideline materials\Social media releases |
| 2. Dissemination/implementation methods\Professional domain\Advertise guideline materials\Media release |
| 2. Dissemination/implementation methods\Professional domain\Advertise guideline materials\Podcast |
| 2. Dissemination/implementation methods\Professional domain\Advertise guideline materials\Submit guideline to guideline clearinghouse/database |
| 2. Dissemination/implementation methods\Professional domain\Advertise guideline materials\App with guideline content |
| 2. Dissemination/implementation methods\Professional domain\Advertise guideline materials\Publish in newsletter |
| 2. Dissemination/implementation methods\Professional domain\Advertise guideline materials\Mass media campaign |
| 2. Dissemination/implementation methods\Professional domain\Advertise guideline materials\Publish in journal |
| 2. Dissemination/implementation methods\Professional domain\Advertise guideline materials\Publish formulary |
| 2. Dissemination/implementation methods\Professional domain\Advertise guideline materials\Website publication(s) |
| 2. Dissemination/implementation methods\Professional domain\Provide additional implementation supporting materials |
| 2. Dissemination/implementation methods\Professional domain\Provide additional implementation supporting materials\Patient pathway |
| 2. Dissemination/implementation methods\Professional domain\Provide additional implementation supporting materials\Toolkit |
| 2. Dissemination/implementation methods\Professional domain\Provide additional implementation supporting materials\Visuals |
| 2. Dissemination/implementation methods\Professional domain\Provide additional implementation supporting materials\Conversation guide |
| 2. Dissemination/implementation methods\Professional domain\Provide additional implementation supporting materials\English version of guideline |
| 2. Dissemination/implementation methods\Professional domain\Provide additional implementation supporting materials\Powerpoint |
| 2. Dissemination/implementation methods\Professional domain\Provide additional implementation supporting materials\Provide presentation with guideline summary |
| 2. Dissemination/implementation methods\Professional domain\Provide additional implementation supporting materials\Clinical decision support tools |
| 2. Dissemination/implementation methods\Professional domain\Provide additional implementation supporting materials\Guideline summary |
| 2. Dissemination/implementation methods\Professional domain\Provide additional implementation supporting materials\Pocket cards summarizing guideline |
| 2. Dissemination/implementation methods\Professional domain\Provide additional implementation supporting materials\Triage guide |
| 2. Dissemination/implementation methods\Professional domain\Provide additional implementation supporting materials\Infographics |
| 2. Dissemination/implementation methods\Professional domain\Provide additional implementation supporting materials\Practice manuals |
| 2. Dissemination/implementation methods\Professional domain\Provide additional implementation supporting materials\Guideline factsheet/summary |
| 3. Implementation evaluation |
| 3. Implementation evaluation\No implementation evaluation |
| 3. Implementation evaluation\Does not evaluate but is positive towards potential/has plans |
| 3. Implementation evaluation\Does not perform evaluation, leaves it to other organizations |
| 3. Implementation evaluation\Difficult to define desired implementation goal |
| 3. Implementation evaluation\Evaluation is a difficult task |
| 3. Implementation evaluation\Difficult to finance implementation evaluation |
| 3. Implementation evaluation\Renewing working methods based on evaluation |
| 3. Implementation evaluation\Outcomes evaluation Appropriate Care Program |
| 3. Implementation evaluation\Process evaluation |
| 3. Implementation evaluation\Process evaluation\Evaluation implementation at staff meeting scientific/professional organization |
| 3. Implementation evaluation\Process evaluation\Visitor numbers guideline materials |
| 3. Implementation evaluation\Process evaluation\Evaluative questions at members meeting |
| 3. Implementation evaluation\Process evaluation\Organization asked hospitals whether they did something with guidelines |
| 3. Implementation evaluation\Process evaluation\Organization asks for guideline feedback but does not receive much |
| 3. Implementation evaluation\Process evaluation\Input from healthcare professionals about guideline |
| 3. Implementation evaluation\Process evaluation\Workgroup that visits and retrieves info from healthcare professionals (focus group) |
| 3. Implementation evaluation\Outcome evaluation |
| 3. Implementation evaluation\Outcome evaluation\Patient organizations do not have capacity to evaluate |
| 3. Implementation evaluation\Outcome evaluation\Research into use of guidelines |
| 3. Implementation evaluation\Outcome evaluation\Guideline developer makes audit & feedback |
| 3. Implementation evaluation\Outcome evaluation\Audit and feedback/benchmarking program national organization |
| 3. Implementation evaluation\Outcome evaluation\Assessment/accreditation through external organization |
| 3. Implementation evaluation\Outcome evaluation\Inspectorate monitors and evaluates |
| 3. Implementation evaluation\Outcome evaluation\Clinical peer review |
| 3. Implementation evaluation\Impact evaluation |
| 3. Implementation evaluation\Court of Audit report about Appropriate Care Program |
| 3. Implementation evaluation\Combination of implementation process, outcomes and/or impact |
| 3. Implementation evaluation\Combination of implementation process, outcomes and/or impact\Appropriate Care Program |
| 3. Implementation evaluation\Combination of implementation process, outcomes and/or impact\Insurers evaluate calamities/cases/letters of insured persons |
| 3. Implementation evaluation\Combination of implementation process, outcomes and/or impact\Insurers monitor through implementation agenda improvement plan |
| 3. Implementation evaluation\Combination of implementation process, outcomes and/or impact\Insurers evaluate based on transparent indicators |
| 3. Implementation evaluation\Combination of implementation process, outcomes and/or impact\Governmental agency monitors and reports impact projects |
| 3. Implementation evaluation\Combination of implementation process, outcomes and/or impact\Impact report based on declaration data to VWS and guideline organizations |
